# An Exploration of Factors Related to Dissemination of and Exposure to Internet-Delivered Behavior Change Interventions Aimed at Adults: A Delphi Study Approach

**DOI:** 10.2196/jmir.956

**Published:** 2008-04-16

**Authors:** Wendy Brouwer, Anke Oenema, Rik Crutzen, Jascha de Nooijer, Nanne K de Vries, Johannes Brug

**Affiliations:** ^3^EMGO InstituteVU University Medical CenterAmsterdamThe Netherlands; ^2^Department of Health Education and Health PromotionSchool for Public Health and Primary Care (Caphri)Maastricht UniversityMaastrichtThe Netherlands; ^1^Department of Public HealthErasmus MCUniversity Medical CenterRotterdamThe Netherlands

**Keywords:** Internet, Internet interventions, health behavior change, dissemination, exposure, Delphi study

## Abstract

**Background:**

The Internet is an attractive medium for delivering individualized, computer-tailored behavior change interventions to large numbers of people. However, the actual numbers of people reached seem to fall behind the high expectations. Insight into factors that determine use of and exposure to these Internet interventions is important to be able to increase the reach and improve exposure.

**Objective:**

The aim was to identify potentially important factors that determine whether adults visit an Internet-delivered behavior change intervention, extend their visit, and revisit the intervention.

**Methods:**

A systematic, three-round Delphi study was conducted among national and international experts from Internet intervention research and practice, e-marketing/e-commerce, Web design, and technical website development. In the first round, 30 experts completed a structured, open-ended online questionnaire assessing factors that were, in their opinion, important for a first visit, an extended visit, a revisit and for effective promotion strategies. Based on the responses in this first questionnaire, a closed-ended online questionnaire was developed for use in the second round. A total of 233 experts were invited to complete this questionnaire. Median and interquartile deviation (IQD) scores were computed to calculate agreement and consensus on the importance of the factors. The factors for which no consensus was obtained (IQD > 1) were included in the third-round questionnaire. Factors with a median score of six or higher and with an IQD ≤ 1 were considered to be important.

**Results:**

Of the 62 experts invited for the first round, 30 completed the questionnaire (48% response rate); 93/233 experts completed the second-round questionnaire (40% response rate), and 59/88 completed the third round (67% response rate). Being motivated to visit an Internet intervention and perceiving the intervention as personally relevant appeared to be important factors related to a first visit. The provision of tailored feedback, relevant and reliable information, and an easy navigation structure were related to an extended visit. Provision of regular new content and the possibility to monitor personal progress toward behavior change were identified as important factors to encourage a revisit. Primarily traditional promotion strategies, like word-of-mouth by family and friends, a publicity campaign with simultaneous use of various mass media, and recommendation by health professionals, were indicated as effective ways to encourage adults to visit an Internet intervention.

**Conclusions:**

This systematic study identified important factors related to the dissemination of and exposure to Internet interventions aimed at adults. In order to improve optimal use of and exposure to Internet interventions, potential users may need to be motivated to visit such an intervention and the information provided needs to be personally relevant. Furthermore, several (technical) aspects of the intervention itself need to be taken into account when developing Internet interventions.

## Introduction

The Internet has dramatically changed the possibilities for communication, including communication about health behavior and behavior change [1]. The Internet is a very attractive medium for the delivery of behavior change interventions since it provides the option of delivering sophisticated versions of individualized, computer-tailored interventions and holds the promise of reaching large numbers of people [2-5]. However, the actual reach of Internet-delivered behavior change interventions seems to lag behind this high expectation [6,7]. Evidence from efficacy trials indicates that actual use of and exposure to the assigned intervention content is low [8,9], and when implemented in real life, exposure rates may be even lower [10,11]. In addition, exposure to the intervention content is not always optimal. It has been demonstrated that it is difficult to sustain visitors’ loyalty to an intervention over an extended period of time [12,13], which may result in premature attrition from a session or in non-use of follow-up sessions. Furthermore, people tend to spend only a limited amount of time assessing the program [14], which makes optimal exposure to the intervention content unlikely. Loyalty to the program over an extended period of time may not be necessary for all Internet interventions or for all people using them since not all Internet interventions require extensive or repeated use of all the offered content [15,16]. However, for all Internet interventions at least some exposure to the intervention content is needed to initiate a process of behavior change. An increase in the number of people reached and improved exposure to Internet-delivered behavior change interventions are needed to be able to achieve optimal implementation of interventions after they have been evaluated to be efficacious [6,9].

The importance of focusing attention not only on intervention efficacy but also on dissemination, reach, and exposure in achieving public health impact is emphasized in the RE-AIM (Reach, Efficacy, Adoption, Implementation, and Maintenance) framework [17]. To be able to improve dissemination and exposure rates of Internet-delivered behavior change interventions, it is important to identify factors that enhance or inhibit these rates since such factors have to be targeted when attempting to improve dissemination and exposure [18]. The present study investigates factors related to dissemination of, use of, and exposure to Internet-delivered behavior change interventions among adults.

Access or use of the Internet is not likely to be a barrier to accessibility of Internet interventions these days since penetration rates of home Internet access and Internet use are high. Various factors have been related to Internet or Internet intervention use, for example, differences in motivation, skills, and availability of computer facilities [9,19]. It has been suggested that to increase the number of first time and extended visits, it is necessary to ensure reliability and credibility of the source or provider of the intervention [20,21]. The information structure has been found to be related to the use of information, with less structured websites tending to prematurely lose visitors [13,22,23]. Also, the amount of detail and elaboration of the information has been related to the length of time people process the intervention information [12]. Furthermore, it has been suggested that a static website that does not change over time may not attract revisits to interventions designed for multiple visits [4]. The use of email to encourage revisiting an intervention seemed to have some effect on revisits, but not on encouraging new users [7,24]. Even though some potentially important determining factors have been suggested in the literature, these factors have not been studied in a systematic way, which is the aim of the present study.

In this study we defined Internet-delivered behavior change interventions (or Internet interventions) to include those interventions that are aimed at the primary prevention of chronic disease by promoting healthful behaviors. Examples are interventions that promote healthful dietary, physical activity, and safe sex practices, discourage alcohol consumption, or encourage smoking cessation or sun protection behavior. Although these are very different topics, similar issues regarding exposure to and use of the content are likely to apply for all these interventions.

Dissemination and use of Internet interventions can be considered a process of diffusion and adoption of the intervention. Therefore, we used the Diffusion of Innovations Theory proposed by Rogers as the theoretical background for this study [18]. According to this model, characteristics of the user, the source (ie, the provider of the intervention), and the innovation (in this case the intervention) are important in the process of dissemination and adoption. Characteristics of the users include personal characteristics, such as gender and age, but also individual cognitions regarding use of Internet interventions, including attitudes, subjective norms, perceived behavioral control, and intention as derived from the Theory of Planned Behavior [25]. Furthermore, perceived possibilities and barriers to use of an intervention may play a role. Potentially important characteristics of the source are the perceived credibility and reliability. Characteristics of the intervention include the complexity (the degree to which the Internet intervention is perceived as difficult to understand and use), the trialability (the degree to which it is possible to experiment with the intervention before adopting it completely), and the relative advantage of the intervention (the degree to which the intervention is perceived to be superior to the idea that it replaces) [14,18]. In this study the term “dissemination” was used for the activities that the developers or providers have to undertake to bring the intervention to the attention of potential users. Dissemination was regarded as being distinct from exposure since the first is more related to activities of providers and the latter to the behavior of potential users. We conceptualized the process of visiting an Internet intervention and being optimally exposed to its educational content as consisting of three distinct phases that are potentially determined by different factors: (1) a first visit, in which a potential user has to decide to go to a website and see what it entails, (2) extending the visit, in which a user has to decide whether to continue his or her visit and be exposed to (part of) the content, and (3) revisiting the Internet intervention, in which the user has to decide to make a return visit to the intervention.

To assess the potential factors related to use of and exposure to Internet interventions, we conducted a three-round Delphi study. The specific aim of this study was to identify the (1) factors that are associated with dissemination of and exposure to (first visit, extended visit, and revisit) Internet interventions aimed at adults, and (2) extent to which experts agree on the importance of these factors.

## Methods

A three-round Delphi study was conducted with international experts from health promotion research, e-marketing/ e-commerce, Web design, and technical website development. A Delphi study is a technique particularly suited for generating ideas about topics on which scientific knowledge is scarce. The technique allows for including experts from all over the world, guarantees anonymity of responses that may make the experts respond more freely, and is aimed at reaching agreement on the important issues [26-28]. The first round of the Delphi study was aimed at identifying potential factors of dissemination, first visit, extended visit, and revisit of an Internet intervention. The aim of the second and third round was to determine the importance and achieve agreement on the importance of the factors identified in the first round. The Delphi study was conducted over the Internet using online questionnaires. It was part of a larger study in which factors of dissemination and use of Internet interventions in adolescents were investigated. In the first round of the study, experts were asked to indicate factors that would be important for adults as well as for adolescents. In the second and third rounds, experts had to provide separate responses for adults and adolescents. The entire Delphi study was carried out within 3 months (October to December 2006). The results regarding adolescents are published elsewhere [29].

### Participants and Procedure

A total of 62 prominent experts in Internet intervention research and practice, e-marketing/e-commerce, Web design, and technical website development from around the world were invited for the first round of the Delphi study. The ratio of experts from each field was set to 30:10:10:10. The highest number of experts was chosen to be from health promotion research and practice since we expected that these experts would have the broadest insight into the effectiveness of dissemination strategies and the factors related to a first visit, an extended visit, and a revisit. Criteria for choosing key experts in the first round were the following: (1) they were first authors of key scientific publications in the area of eHealth and eHealth promotion, and (2) they had written multiple scientific articles regarding this topic. People were also included if they were active members of editorial boards of leading journals in health promotion and the Internet and had published in these areas or journals. Representatives of e-marketing/e-commerce and ICT (information and communication technology) companies (eg, Web designers and developers) were selected on the basis of publications, our own network, and by asking the responders to provide names of other experts in their field.

This list of experts was extended to 233 persons (aim was 250) to be invited for participation in the second round of the study. The criterion for selection was being first author of a scientific paper or abstract on the topic of Internet interventions. Names of first authors were retrieved through a literature search in PubMed, PsycINFO, and Web of Science (between 2000 and 2006), and first authors of abstracts published in proceedings of relevant national and international conferences (eg, Society for the Internet in Medicine [MEDNET 2005 and 2006] and International Society for Behavior Nutrition and Physical Activity [ISBNPA 2004-2006]) were added to the list. Experts from the field of e-marketing/e-commerce and ICT were mainly found through our own network and by referral from experts in the first round. The experts who responded in the second round (n = 88) were invited to participate in the third round.

The experts were invited to participate in the study and each subsequent round by means of an email. In this email, the purpose and procedure of the Delphi study was explained and a link to the questionnaire was provided. Invitees were reminded once by email to complete the first-round questionnaire and twice to complete the second- and third-round questionnaires. The questionnaires were pre-tested by experts in the fields of health promotion research and e-marketing.

### Measurements

#### First Round

The first-round questionnaire was a structured questionnaire with an open-ended answer format. Participants were asked to list all the factors that, according to their expertise, (1) are essential for successful dissemination of Internet interventions, (2) determine whether a person will visit an intervention for the first time, (3) determine whether a person will stay long enough on a website to meaningfully engage in the educational content, and (4) determine whether a person will revisit a website. A sample question was “What are, according to your expertise, factors that determine whether a person will visit an Internet-delivered behavior change intervention for the first time?” The respondents were asked to suggest factors related to the user, the source, the Internet intervention itself, the physical and social environment, and any other important factors. The questionnaire started with a definition of all concepts used (eg, what we defined as factors, Internet-delivered interventions, behavioral topics addressed in these interventions, and dissemination).

#### Second Round

The second-round questionnaire had a closed-ended answer format and included all the unique factors that had been mentioned by the experts in the first round, except for those that were general health education principles not unique to Internet interventions (eg, the intervention is based on scientific knowledge, the information should be understandable) since these are basic principles for state of the art health communication interventions for which no rating of importance and consensus is needed. The questionnaire consisted of 82 statement items (see the Multimedia Appendix) presenting factors related to the (potential) visitor, the source, and the Internet intervention itself for a first visit, extended visit, revisit and for dissemination. The experts were asked to indicate how important they thought each of the factors were on a 7-point Likert scale (1 = not important, 7 = extremely important) for adults and adolescents separately. Apart from determinants of dissemination, the experts in the first round mentioned many factors that were, in fact, ways to promote Internet interventions. Therefore, we included a list with 23 strategies for promoting an Internet intervention. The experts were asked to choose the five strategies they thought were most successful for promoting an intervention among adults. This list of promotion strategies appeared in random order for each of the respondents.

#### Third Round

The third-round questionnaire contained the items (48 in total, see the Multimedia Appendix) of the second-round questionnaire for which no consensus was obtained (interquartile deviation [IQD] > 1). The answering scale for each item now included information on the median score and IQD for that item as determined in the second-round questionnaire. The experts were asked to re-rate their answers on the same 7-point Likert scale in the light of this new information.

### Data Analysis

All the responses to the first-round questionnaire were listed, and similar responses were grouped together to reduce the number of factors. The remaining list of potentially important factors was included in the questionnaire for the second and third round, except for the factors that were general health education principles.

In the second round, following the standards for analyzing data from a Delphi study, the median scores were calculated to determine agreement on the importance of the statements. Also, the IQDs were calculated to determine consensus among the experts on the importance of the statements [26,30]. On a 7-point Likert scale, an IQD ≤ 1 can be considered as good consensus and means that more than 50% of all opinions fall within one point on the scale [28]. Items with a median ≥ 6 (very or extremely important) and an IQD ≤ 1 were considered as important factors. The dissemination strategies were analyzed by means of multiple response analysis.

In the third round, median scores and IQDs were calculated for the items included in the third-round questionnaire. SPSS 11.0 (SPSS Inc, Chicago, IL, USA) was used for all the statistical analyses.

## Results

### Participants and Response Rates

In total, 30 of the 62 experts we approached completed the questionnaire in the first round (48% response rate; Table 1). Participants were primarily from health promotion institutes (64% response rate) and health promotion research (50% response rate); 93/233 respondents completed the second-round questionnaire (40% response rate), and 59/88 completed the third-round questionnaire (67% response rate). Three participants resigned from participation in the third round due to time constraints, and two could not be contacted again since they had not provided contact details in the previous questionnaire. Reasons for nonparticipation and dropout of the other experts are not known, although some reported lack of time or interest.

**Table 1:** Response rates in the Delphi study

Discipline	First Round			Second Round			Third Round		
	No. Invited	No. Responded	%	No. Invited	No. Responded	%	No. Invited	No. Responded	%
Health promotion research	32	16	50	155	65	42	62	41	66
Health promotion institutes	11	7	64	20	10	50	10	8	80
e-Marketing and communication	9	3	33	24	6	25	6	4	67
Technical implementation	10	4	40	34	10	29	10	6	60
Unknown	–	–	–	–	2	–	–	–	–
Total	62	30	48	233	93	40	88	59	67

### Measurements

#### First Round

All factors unique for Internet interventions identified in the first round are listed in the Multimedia Appendix. This list is composed of factors that were mentioned by individual experts (eg, using modular approach, an enjoyable and rewarding experience in the first visit), as well as factors that were brought up by several of the experts (eg, tailored/individualized content, word-of-mouth by family and friends, the credibility of the source). More factors were mentioned for a first visit and an extended visit than for a revisit. The factors mentioned under dissemination were mainly ways to promote an intervention, such as word-of-mouth, commercials on TV and radio, and email.

#### Second Round

With respect to the first visit, 4 of 17 items pertaining to the potential visitor (sufficient Internet skills, experience with using the Internet, motivation to visit the intervention, perceived relevance of the intervention) and 2 of 9 items pertaining to the Internet intervention (instant use, easy navigation structure) had a median score ≥ 6 (Table 2). Consensus was reached for three of these items.

Regarding an extended visit, 5 of 9 items related to the visitor (eg, wants to improve behavior, experiences the use as rewarding, appreciates tailored feedback), 0 related to the source, and 12 of 23 items related to the Internet intervention (eg, displays personal progress, provides brief registration procedure, free of charge) had a median score ≥ 6 (Table 2). Consensus was reached for 10 of these items.

With respect to revisiting an intervention, 4 of 5 items regarding the visitor (receiving a reminder, committed to revisit, wants to improve behavior, positive experience with previous visit) and 5 of 10 items pertaining to the Internet intervention (new content, monitoring progress, experienced previous visit as easy, rewarding, and enjoyable) had a median score ≥ 6 (Table 2). Consensus was reached for all these items, indicating that the majority of experts agreed that these were important factors for revisiting.

None of the strategies for dissemination had a median score ≥ 6 (see the Multimedia Appendix).

Overall, consensus (IQD ≤ 1) was reached for 34 items in the second round. Most items that reached consensus were related to revisiting an intervention (10 of 15 items). The least consensus was achieved for dissemination of interventions (1 of 7 items).

**Table 2:** Results of the Delphi study per item (second and third round) with a median score ≥ 6 (full list of results including items with lower scores can be found in the Multimedia Appendix)

Questionnaire Item	Second Round			Third Round		
	No.	Median^†^	IQD	No.	Median^†^	IQD
**I. How important do you think each of the following factors are in determining whether a person will make a first visit to an Internet-delivered behavior change intervention?**						
A. Whether the potential visitor						
- has sufficient skills to use the Internet	89	6	1.5	59	6	1
- has experience with using the Internet	88	6	1	–^*^	–	–
- is motivated to visit a behavior change intervention provided through the Internet	88	6	1	–	–	–
- perceives the Internet intervention as relevant for himself/herself	84	6	1	–	–	–
						
B. Whether the Internet intervention						
- can be used instantly without downloading special software by the potential visitor	83	6	2	56	6	0
- has a navigation structure that appears to be easy to use at first sight	83	6	2	56	6	0
						
**II. How important do you think each of the following factors are in determining whether a person will stay on an Internet-delivered behavior change intervention long enough to actively engage in and process the educational content provided in the intervention?**						
A. Whether the visitor						
- knows in advance how long it will take to go through the whole intervention	80	6	2	56	6	1
- wants to improve his/her behavior in relation to the topic of the Internet intervention	80	6	1	–	–	–
- perceives the topic and content of the entire Internet intervention as being personally relevant	79	6	2	56	6	0
- experiences the use of the Internet intervention as rewarding	80	6	1	–	–	–
- likes receiving (tailored) feedback on the answers he/she provided on questions	80	6	2	56	6	1
						
C. Whether the Internet intervention						
- displays personal progress through the program (eg, progress bar, page numbers)	78	6	1	–	–	–
- provides the opportunity for a visitor to stop at any moment and to proceed at a later time	79	6	1	–	–	–
- has an aim that is clear to the visitor	79	6	1	–	–	–
- provides information that appears reliable to the visitor	78	6	1	–	–	–
- provides information that is easy to understand for the visitor	79	6	1	–	–	–
- provides information that is perceived to be useful for the visitor to help him/her in changing behavior	77	6	2	56	6	0
- has a tone of voice that is appealing to the visitor	78	6	1	–	–	–
- has an easy-to-follow navigation structure	78	6	2	56	6	0
- provides tailored feedback	77	6	1	–	–	–
- provides tailored feedback which is perceived as relevant to the visitor	77	6	1	–	–	–
- provides behavior change information that seems achievable to the visitor	77	6	2	56	6	0
- can be used free of charge	77	6	2	55	6	0
						
**III. How important do you think each of the following factors are in determining whether a person will revisit an Internet-delivered behavior change intervention?**						
A. Whether the visitor						
- receives a reminder to revisit the Internet intervention	76	6	1	–	–	–
- is committed to revisiting the Internet intervention	76	6	1	–	–	–
- wants to improve his/her behavior in relation to the topic of the Internet intervention	76	6	1	–	–	–
- had a positive experience with the previous visit to the Internet intervention	76	6	1	–	–	–
						
B. Whether the Internet intervention						
- provides new content on a regular basis	76	6	1	–	–	–
- provides the possibility for a visitor to monitor his/her progress in changing behavior	76	6	1	–	–	–
- has previously been experienced as easy to use by the visitor	76	6	1	–	–	–
- has previously been experienced as rewarding by the visitor	76	6	1	–	–	–
- has previously been experienced as enjoyable by the visitor	76	6	1	–	–	–

^*^Dashes indicate that consensus was obtained on the item in the second round and, for that reason, was excluded from the third-round questionnaire.

^†^All items were scored 1-7 on a 7-point Likert scale.

The ways to disseminate Internet interventions that were indicated most often were word-of-mouth by family and friends (58.1%), a publicity campaign with the simultaneous use of various mass media (58.1%), and recommendation by health professionals (52.7%; Table 3).

**Table 3:** Strategies of Internet intervention dissemination (N = 74)

Dissemination Strategy	No.	%
Word of mouth (eg, by friends and family)	43	58.1
Publicity campaign with simultaneous use of various mass media	43	58.1
Health professionals (eg, general practitioner, physical therapist)	39	52.7
TV and radio programs (eg, talk shows, consumer programs)	31	41.9
Commercials on TV and radio	28	37.8
Articles in magazines and newspapers	25	33.8
Links to the Internet intervention at other websites	20	27.0
Involvement of people who belong to the target group	20	27.0
Advertisements on websites visited by the target group	19	25.7
Face-to-face contact	18	24.3
Email	17	23.0
Banners of the Internet intervention on other websites	14	18.9
Nonmedical professionals (eg, worksite health promoter)	14	18.9
Advertisements in magazines and newspapers	12	16.2
Advertisements on relevant products (eg, cigarette packs, milk cartons)	10	13.5
Free publicity (eg, postcards, brochures, bulletin board postings in libraries or hospitals)	9	12.2
Use of virtual guides to direct people to the Internet intervention (eg, in chat boxes)	8	10.8
Telephone calls	7	9.5
Forums on the Internet	4	5.4
Other ICT channels (eg, MSN Messenger, AOL Instant Messenger)	3	4.1
Distribution of flyers at exhibitions and other public events	2	2.7
Distribution of flyers door-to-door	1	1.4
SMS (Short Message Service)	0	0.0

#### Third Round

The median scores of the items included in the third-round questionnaire did not differ from the second round. Consensus was achieved for 45 of the 48 items (IQD ≤ 1; see Table 2). No consensus was achieved for positive expectations of behavior change interventions delivered through the Internet (relating to first visit), whether the user has to provide sensitive information, or the option of a trial before starting the intervention (related to extended visit). These three factors had a median score < 6.

## Discussion

### Summary of Findings

This Delphi study is among the first systematic explorations of potentially important factors related to the dissemination of and exposure to Internet-delivered behavior change interventions. The study is unique in its focus on factors related to a first visit, an extended visit, and a revisit and by taking into account the characteristics of the potential users (in this case, adults), the source, and the intervention itself. In particular, factors related to the potential user, such as motivation and perceived personal relevance, were identified as important factors (median score > 6; IQD ≤ 1) related to a first visit. With regard to an extended visit (ie, staying on the intervention long enough to meaningfully process some of the content), many more factors related to the intervention itself were identified as important. The intervention needs to provide tailored feedback and relevant and reliable information and be clear and easy to use. The experience with the intervention in the previous visit, the inclination to change the behavior targeted in the intervention, the provision of new content, and being reminded to visit the intervention were regarded as important factors for a revisit. Apart from the factors that were rated as very important or extremely important, most of the other factors that came out of the first round reached consensus and were rated as somewhat important or important (median score 4-5). This means that these factors (listed in the Multimedia Appendix) also need to be taken into account when attempting to improve use and exposure to Internet interventions.

### Interpretation of Findings

The existing knowledge on factors that enhance or inhibit optimal use of and exposure to an Internet intervention mainly relate to characteristics of the intervention itself. In this Delphi study we used the Diffusion of Innovations Theory [18] as a theoretical framework, and therefore, we also considered characteristics of the user and the source as potentially important factors associated with adoption. In contrast to previous studies, credibility and reliability of the source were not identified as very important factors for visiting an Internet intervention or extending a visit [20,21]. With respect to characteristics of the potential users, motivation to visit the intervention and perceived personal relevance of the intervention were identified as important factors. The finding that motivation is an important factor is intuitive since visiting an Internet intervention for the first time, extending the visit, and revisiting the intervention can be considered as specific behaviors that can be explained by the Theory of Planned Behavior [18]. According to this theory, motivation is the determinant most proximal to behavior. The present study did not, however, provide information about factors underlying the motivation to visit an Internet intervention, such as attitudes, subjective norms, or perceived behavioral control [18]. This is possibly due to the breadth of topics addressed in this study or that the study was performed among experts and not among the actual users of Internet interventions. Nevertheless, motivating people to visit an Internet intervention seems to be important.

The provision of personalized feedback seems to be a key element related to an extended visit to an Internet intervention. This finding underlines what has been previously suggested in the literature. Computer tailoring has been identified as a very promising health education technique and the Internet, as a suitable medium for delivery of computer-tailored interventions [31,32]. Furthermore, if the computer-tailored information is iterative and provides new information and information about the users’ progress, it might also encourage people to revisit the intervention [3,4,33-35].

Not only are motivation and personal feedback important, but the way in which the information is presented was also identified as an important factor for extending a visit and revisiting an Internet intervention. The navigation structure of the intervention must appear attractive and easy to use, as has been stressed before by Danaher et al [22]. Also, the intervention itself must look attractive at the very fist encounter (within 50 ms since an opinion about visual attractiveness is formed that quickly) [23]. Furthermore, the information obtained needs to be experienced as enjoyable and rewarding, but visitors must also find it usable and easy to understand [36].

An important factor to encourage people to revisit an Internet intervention that is designed for multiple visits is the provision of new content on a regular basis as there may be no need to return if the website does not change over time [4]. To make a revisit attractive, different aspects can be added to make the intervention less static, such as providing iterative tailored feedback or indicating what can be expected in a next visit. Another way to attract people to revisit the intervention is by reminding them, for example through email.

The communication channels most often indicated as potentially effective dissemination strategies were the more traditional channels such as word-of-mouth by family and friends [12], a publicity campaign with simultaneous use of various mass media, and recommendation by health professionals. Also, “old fashion” promotion strategies such as a publicity campaign, TV and radio commercials and programs, and articles in newspapers were seen as effective. The more novel channels, such as SMS, instant messaging, and banners on other websites, were hardly selected as important channels for dissemination.

### Limitations

There are some limitations to the study that need to be mentioned. We tried to incorporate experts from several disciplines as well as technical and marketing backgrounds. However, experts from technical and marketing backgrounds were underrepresented and responded less in the second and third round. Thus, the factors that were identified are more strongly based on the expert opinion of health educators and health promoters, and important factors from the technical and marketing field may have been missed. However, consensus was reached for most of the factors, which indicates that there were hardly any differences in the responses of experts from the various fields. Response rates in the various rounds ranged between 40% and 67%. Even though these response rates seem quite low, they are comparable to those found in other Delphi studies [26]. The low response rates may be due to the time investment that was required from the experts. They were asked to complete two or three questionnaires within 3 months. The low response rates may have resulted in the inclusion of a select group of experts, which may have introduced bias. We expect, however, that potential bias due to this selected sample is limited since the experts who participated provided a large variety of potentially important factors and saturation seemed to have been reached. Nevertheless, we cannot completely rule out the possibility that potentially important factors may have been missed. Most nonrespondents did not give a reason for not responding, but those who did mostly reported lack of time.

The Diffusion of Innovations Theory [18] and, within that, the Theory of Planned Behavior [25] that we used as a framework may not have been a complete fit for the present study and may have prevented us from looking at other potentially important factors. Another limitation may be that we tried to get information about various aspects of the process of visiting and revisiting an intervention. This breadth of topics may have been at the expense of the depth of information. The fact that mainly general factors were identified, such as “motivation” or “a rewarding experience,” and not factors that constitute motivation or a rewarding experience may be an indication of this. However, the aim of the present study was to gain a broad insight.

The results of the present study provide information about important factors for a first visit, extended visit, and a revisit that apply to most Internet-delivered behavior change interventions but that are not really intervention specific. Furthermore, not all factors identified in the present study may be equally applicable to all Internet interventions aimed at the primary prevention of chronic disease. That is because there is huge variety in the type of Internet intervention (low-intensity interventions without follow-up to very intensive interventions with up to 1 year follow-up), behavior targeted in the intervention, behavior change strategies applied, and so on. Therefore, for each intervention, the most applicable factors have to be chosen.

### Conclusion

In this systematic exploration of potentially important factors determining whether adults visit an Internet-delivered behavior change intervention for the first time, extend a visit, and revisit the intervention, a number of factors were identified that can be taken into account when developing new Internet interventions. Further determinant research is needed to confirm the findings of this study and to identify important exposure-related factors from the perspective of the potential users.

## Multimedia Appendix

Results of the Delphi study per item (second and third round)

## Abbreviations

ICT: information and communication technology
IQD: interquartile deviation
SMS: Short Message Service
