# Supplementary material for: An Exploration of Factors Related to Dissemination of and Exposure to Internet-Delivered Behavior Change Interventions Aimed at Adults: A Delphi Study Approach
Source: J Med Internet Res. 2008 Apr 16;10(2):e10. doi: 10.2196/jmir.956 (PMC2483924; doi:10.2196/jmir.956)
Supplement: Supplementary file 1 [file jmir_v10i2e10_app1.pdf]

Multimedia Appendix

Brouwer W, Oenema A, Crutzen R, Nooijer Jde, Vries NKde, Brug J
An Exploration of Factors Related to Dissemination of and Exposure to Internet-Delivered Behavior Change Interventions Aimed at Adults: A Delphi Study Approach
J Med Internet Res 2008;10(2):e10
http://www.jmir.org/2008/2/e10/
http://dx.doi.org/10.2196/jmir.956

Results of the Delphi study per item (second and third round)

Questionnaire item[a]	Second round			Third round		
	N	Mdn[b,c]	IQD[c]	N	Mdn[b,c]	IQD[c]
**I. How important do you think each of the following factors are in determining whether a person will make a first visit to an Internet delivered behavior change intervention?**						
**A. Whether the potential visitor**						
1. has sufficient skills to use the Internet	*89*	*6*	*1.5*	*59*	*6*	*1*
2. has experience with using the Internet	*88*	*6*	*1*	--	--	--
3. has access to the Internet at a private location (e.g. home work)	89	5	1	--	--	--
4. has positive expectations of behavior change interventions delivered through the Internet	89	5	2	57	5	2
5. is motivated to visit a behavior change intervention provided through the Internet	*88*	*6*	*1*	--	--	--
6. wants to improve his/her behavior in relation to the topic of the Internet intervention	88	5	2	58	5	1
7. is curious about what the Internet intervention has to offer	84	5	1	--	--	--
8. is willing to spend time on visiting an Internet intervention	84	5	1	--	--	--
9. has a positive attitude regarding the use of behavior change interventions delivered through the Internet	83	4	1	--	--	--
10. receives an incentive for visiting the Internet intervention	84	3	3	57	3	1
11. is referred to the Internet intervention by a health professional (e.g. GP physical therapist dietician)	83	5	2	56	5	1
12. gets a positive recommendation about the Internet intervention by word of mouth (e.g. friends family)	84	5	1	--	--	--
13. receives a reminder to visit the Internet intervention	85	5	2	57	5	1
14. perceives the Internet intervention as relevant for him/herself	*84*	*6*	*1*	--	--	--
15. knows that the Internet intervention is effective	85	5	2	57	5	1
16. perceives the source (the organization that provides the intervention) of the Internet intervention as credible	85	5	1	--	--	--
17. perceives the source (the organization that provides the intervention) of the Internet intervention as reliable	85	5	2	57	5	1
**B. Whether the Internet intervention**						
1. has an easy to remember domain name (URL)	83	5	3	56	5	1
2. has a high search engine ranking (e.g. Google Yahoo! AltaVista)	83	5	3	55	5	1
3. can be used with all types of Internet connections like dial-up DSL cable and fiberglass	83	5	3	56	5	1
4. can be used instantly without downloading special software by the potential visitor	*83*	*6*	*2*	*56*	*6*	*0*
5. has an attractive interface at first sight	83	5	1	--	--	--
6. has a navigation structure that appears to be easy to use at first sight	*83*	*6*	*2*	*56*	*6*	*0*
7. is created by experts in health behavior change	82	4	3	56	4	1
8. is endorsed by health professionals	82	5	1.5	56	5	0
9. is based on scientific knowledge	83	5	2	56	5	1
**II. How important do you think each of the following factors are in determining whether a person will stay on an Internet delivered behavior change intervention long enough to actively engage in and process the educational content provided in the intervention?**						
**A. Whether the visitor**						
1. can associate him/herself with the look and feel of the Internet intervention	80	5	2	56	5	1
2. knows in advance how long it will take to go through the whole intervention	*80*	*6*	*2*	*56*	*6*	*1*
3. has to provide sensitive information to register (e.g. home address)	78	5	2	54	5	2
4. wants to improve his/her behavior in relation to the topic of the Internet intervention	*80*	*6*	*1*	--	--	--
5. perceives the topic and content of the entire Internet intervention as being personally relevant	*79*	*6*	*2*	*56*	*6*	*0*
6. experiences the use of the Internet intervention as rewarding	*80*	*6*	*1*	--	--	--
7. experiences the use of the Internet intervention as challenging	80	4	2	56	4	1
8. experiences the use of the Internet intervention as enjoyable	80	5	1.75	56	5	0
9. likes receiving (tailored) feedback on the answers he/she provided on questions	*80*	*6*	*2*	*56*	*6*	*1*

**B. Whether the source of the Internet intervention (the organization that provides the intervention)**						
1. is identifiable as credible by the visitor (e.g. through a logo link to the website of the source or a disclaimer etc.)	80	5	2	56	5	1
2. is identifiable as reliable by the visitor (e.g. through a logo link to website of the source or a disclaimer etc.)	78	5	2	56	5	0
**C. Whether the Internet intervention**						
1. provides the option of a trial before starting for real	78	4	3	56	4	2
2. uses visual materials (e.g. graphs videos pictures)	79	5	2	56	5	0
3. provides interactive features (e.g. tests forums games etc.)	79	5	1	--	--	--
4. displays personal progress through the program (e.g. progress bar page numbers)	*78*	*6*	*1*	--	--	--
5. provides the opportunity for a visitor to stop at any moment and to proceed at a later time	*79*	*6*	*1*	--	--	--
6. uses a virtual guide to guide a visitor through the Internet intervention	79	4	3	56	4	1
7. is attractive for the visitor to use	79	5	1	--	--	--
8. has a brief registration procedure (e.g. the registration of login name and password)	79	5	3	56	5	1
9. has an aim that is clear to the visitor	*79*	*6*	*1*	--	--	--
10. provides testimonials of successes of previous visitors	79	5	3	56	5	1
11. provides information that appears reliable to the visitor	*78*	*6*	*1*	--	--	--
12. provides information that is easy to understand for the visitor	*79*	*6*	*1*	--	--	--
13. provides information that is perceived to be useful for the visitor to help him/her in changing behavior	*77*	*6*	*2*	*56*	*6*	*0*
14. has a tone of voice that is appealing to the visitor	*78*	*6*	*1*	--	--	--
15. has an easy to follow navigation structure	*78*	*6*	*2*	*56*	*6*	*0*
16. provides brief textual information (i.e. does not involve a lot of reading)	78	5	1	--	--	--
17. uses a short questionnaire for providing tailored feedback	77	5	2	56	5	1
18. does not take much time to complete entirely	77	5	1.5	56	5	1
19. provides tailored feedback	*77*	*6*	*1*	--	--	--
20. provides tailored feedback which is perceived as relevant to the visitor	*77*	*6*	*1*	--	--	--
21. provides tailored feedback in sequence of brief questionnaires and brief feedback sections	76	5	2	56	5	1
22. provides behavior change information that seems achievable to the visitor	*77*	*6*	*2*	*56*	*6*	*0*
23. can be used free of charge	*77*	*6*	*2*	*55*	*6*	*0*
**III. How important do you think each of the following factors are in determining whether a person will revisit an Internet delivered behavior change intervention?**						
**A. Whether the visitor**						
1. receives a reminder to revisit the Internet intervention	*76*	*6*	*1*	--	--	--
2. is committed to revisiting the Internet intervention	*76*	*6*	*1*	--	--	--
3. wants to improve his/her behavior in relation to the topic of the Internet intervention	*76*	*6*	*1*	--	--	--
4. has a positive experience with the previous visit to the Internet intervention	*76*	*6*	*1*	--	--	--
5. has a chance to receive an incentive by revisiting the Internet intervention	76	5	2	56	5	1
**B. Whether the Internet intervention**						
1. provides new content on a regular basis	*76*	*6*	*1*	--	--	--
2. provides the possibility for a visitor to monitor his/her progress in changing a behavior	*76*	*6*	*1*	--	--	--
3. includes the option for the visitor to communicate with others (e.g. chat rooms blogs forums)	76	5	1	--	--	--
4. makes clear what the visitor can expect during a revisit (e.g. by a preview)	75	5	2	56	5	0
5. provides the possibility to post questions for professionals (e.g. dietician GP physical therapist)	76	5	2	56	5	1
6. uses a modular approach in which a new visit provides access to the next module	76	4.5	2.75	55	5	1
7. has previously been experienced as easy to use by the visitor	*76*	*6*	*1*	--	--	--
8. has previously been experienced as rewarding by the visitor	*76*	*6*	*1*	--	--	--
9. has previously been experienced as challenging by the visitor	76	4	2	55	4	1
10. has previously been experienced as enjoyable by the visitor	*76*	*6*	*1*	--	--	--

IV. How important do you think each of the following strategies are in achieving successful dissemination of Internet

**interventions?**						
1. Provide the Internet intervention in a controlled setting e.g. worksites (for adults) or school curriculum (for adolescents)	74	4	2	56	4	1
2. Instruction of executives (e.g. workshops for employees or nurses) before using it in a controlled setting	74	5	2.25	55	5	1
3. Embed the Internet intervention in the social context (e.g. at a sports club or at work)	73	5	3	56	5	1
4. Give the Internet intervention an appealing name	74	5	1	--	--	--
5. Assure a high search engine ranking of the Internet intervention (in e.g. Google Yahoo! AltaVista)	75	5	3	56	5	1
6. Embed the Internet intervention in other (health) programs	74	5	2	56	5	1
7. Co-operate with commercial partners (e.g. supermarkets related products soaps) for promotion of the Internet intervention	74	5	3	55	5	1

[a] All items of the questionnaire are included. Dashes indicate that consensus was obtained on the item in the second round and for that was excluded from the third round questionnaire. Items on which consensus (IQD 1) was obtained and which had a median score 6 are printed in italics

[b] All items ranged from 1 to 7

[C] Mdn, median scores; IQD, interquartile deviations
